# Genetics of longevity. data from the studies on Sicilian centenarians

**DOI:** 10.1186/1742-4933-9-8

**Published:** 2012-04-23

**Authors:** Carmela R Balistreri, Giuseppina Candore, Giulia Accardi, Manuela Bova, Silvio Buffa, Matteo Bulati, Giusi I Forte, Florinda Listì, Adriana Martorana, Marisa Palmeri, MariaValeria Pellicanò, Loredana Vaccarino, Letizia Scola, Domenico Lio, Giuseppina Colonna-Romano

**Affiliations:** 1 Department of Pathobiology and Medical and Forensic Biotechnologies, University of Palermo, Corso Tukory 211, Palermo 90134, Italy; 2 Department of Internal Medicine II, Center for Medical Research, University of Tübingen, Tübingen Ageing and Tumor Immunology group, Waldhörnlestr. 22, Tübingen 72072, Germany

**Keywords:** Immune system, Genetics, Pro/anti-inflammatory polymorphisms, Epigenomics

## Abstract

The demographic and social changes of the past decades have determined improvements in public health and longevity. So, the number of centenarians is increasing as a worldwide phenomenon. Scientists have focused their attention on centenarians as optimal model to address the biological mechanisms of "successful and unsuccessful ageing". They are equipped to reach the extreme limits of human life span and, most importantly, to show relatively good health, being able to perform their routine daily life and to escape fatal age-related diseases, such as cardiovascular diseases and cancer. Thus, particular attention has been centered on their genetic background and immune system. In this review, we report our data gathered for over 10 years in Sicilian centenarians. Based on results obtained, we suggest longevity as the result of an optimal performance of immune system and an over-expression of anti-inflammatory sequence variants of immune/inflammatory genes. However, as well known, genetic, epigenetic, stochastic and environmental factors seem to have a crucial role in ageing and longevity. Epigenetics is associated with ageing, as demonstrated in many studies. In particular, ageing is associated with a global loss of methylation state. Thus, the aim of future studies will be to analyze the weight of epigenetic changes in ageing and longevity.

## Introduction

### Data from centenarian offspring

As well known, life expectancy is a familial trait and longevity is determined by different factors. In particular, the environmental milieu and genetic background play a central role. As demonstrated by many epidemiological studies, family members of long-lived subjects have a significant survival advantage compared to general population. In this context, the study of centenarian offspring (CO), a group of healthy elderly people with a familiar history of longevity, might help gerontologists to better identify the correlation between genetic profile and hope of a healthy ageing. Previous studies have reported that CO, like their centenarian parents, have genetic and immune system advantages, which reflect a minor risk to develop major age-related diseases, such as cardiovascular diseases, hypertension or diabetes mellitus as well as cancer [1,2]. The lower cardiovascular disease risk in CO suggests the probability that CO have some protective factors against atherosclerosis, such as a good lipid profile. Male CO have higher plasma HDL-C levels and lower plasma LDL-C levels. Since lipid profile is directly correlated to atherosclerotic cardiovascular diseases, this metabolic feature could preserve CO both to develop these diseases and, as consequence, to reach a healthy ageing and longer survival [3]. Furthermore, Rose et al. [4] reported that centenarians and CO show significantly higher levels of heteroplasmy in mtDNA control region than controls, a favorable condition for longevity.

In these last years, some researchers have speculated about the distinctive immunological profile of offspring enriched for longevity respect to the immunological features of coeval elderly. The cytomegalovirus (CMV) is one of the most common viruses that affect elderly people. Many evidences have shown that CMV infection may influence the T cell subset distribution, having an essential role in immunosenescence [5-7]. CMV infection is strongly related to both a reduction of CD8^+^CD45^+^CCR7^+^CD27^+^CD28^+ ^naïve T cells and to a contemporarily increase of CD8^+^CD45RA^-^CCR7^-^CD27^-^CD28^- ^late differentiated effector memory and CD45RA-re-expressing T cells. These parameters are considered typical of immunosenescence in elderly. Recently, it has been demonstrated that CMV-seropositive offspring of long-lived people don't show the age-associated decrease of naïve T cells. On the other hand, memory T cell subsets above described do not increase in offspring of long-lived families, differently from that observed in age-matched controls [8]. It has been also demonstrated that CMV-seropositive offspring of long-lived people have reduced levels of CD8+ T cells expressing CD57 and KLRG1, sometimes referred as "marker of senescence", when compared to their CMV-infected age-matched controls. The reduction of effector memory T cells lacking the expression of CD27 and CD28 and expressing CD57 and KLRG1, observed in CMV-infected offspring could explain their high proliferative response against CMV. The CMV-seropositive offspring have also shown significantly lower CRP levels compared to their CMV-seropositive age-matched controls that could be related to a lower pro-inflammatory status [8].

During ageing, B cell compartment also shows significant modifications in numbers and functions [9-12]. In fact, advanced age is per se a condition characterized by lack of B clonotypic immune response to new extracellular pathogens. In any event, data are suggesting that the loss of naive B cells could represent a hallmark of immunosenescence [13]. On the other hand, a B cell population lacking of both IgD and CD27 resulted increased in healthy elderly [14]. We have suggested that this IgD^-^CD27^- ^B cell subset is a population of memory B cells lacking CD27, a typical memory marker, likely considered a late memory exhausted B cell subset (Table 1) [14-16]. This population resulted also increased in active Lupus patients [17], in healthy subjects challenged with respiratory syncitial virus [18], and in HIV patients [19]. CO don't show the typical naïve/memory B cell shift observed in elderly. Although a decreased B cell count was observed in CO and their age-matched controls, it has been demonstrated that naïve B cells (IgD^+^CD27^-^) were more abundant and DN B cells (IgD^-^CD27^-^) were significantly decreased, as looked similarly in young people [20]. This B cells distribution in CO could suggest that antigenic load or inflammatory environment play a central role in exhaustion of the B cell branch. It is well documented that the quality and the size of the humoral immune response declines with age [15,21-26]. This change is characterized by lower antibody responses and decreased production of high affinity antibodies. The evaluation of IgM secreted in CO serum shows that the values are within the range of the levels observed in young subjects [20]. In this way, CO could have a bigger advantage to fight against new infections and appropriately respond to vaccinations, giving them a selective advantage for longevity in healthiness.

**Table 1:** Main modifications of B cells and B cells products in elderly human observed in our laboratory

B cells or B cells products	Changes	References
Total B cells (percentage)	↓	[9]
CD19^+^CD5^+ ^B1 cells (percentage and absolute number)	↓	[10]
IgG, IgA	↑	[11]
IgM, IgD	↓	[11]
IgE	=	[11]
Autoantibodies	↑	[12]
Naive (IgD^+^CD27^-^)	↓	[13]
DN (IgD^-^CD27^-^)	↑	[14-16]

In conclusion, individuals genetically enriched for longevity possess immune different signatures respect to those of the general population (Table 2). This suggests the idea of the "familiar youth" of the immune system. In addition, the lower pro-inflammatory status in CMV-infected offspring of long-lived people might represent an optimal advantage for healthy longevity and against mortality associated to major age-related diseases.

**Table 2:** Cellular and humoral immune modification in offspring from longevity families compared to their AM controls

T and B cell Phenotypes and Products	Changes	References
Naïve T cells (CD3^+^CD8^+^CD45RA^+^CCR7^+^CD27^+^CD28^+^)	Increase	[8]
Late differentiated effector memory T cells (CD3^+^CD8^+^CD45RA^-^CCR7^-^CD27^-^CD28^-^)	Decrease	[8]
TEMRA (CD3^+^CD8^+^CD45RA^+^CCR7^-^CD27^-^CD28^-^)	Decrease	[8]
Naïve B cells (IgD^+^CD27^-^)	Increase	[20]
Double Negative B cells (IgG^+^/IgA^+^IgD^-^CD27^-^)	Decrease	[20]
Serum IgM	Increase	[20]

### Gender and longevity

A characteristic enigma of longevity is the gender and the social phenomenon of "*feminization of old age*". The demographic and social changes of the past decades, responsible for longevity and the improvements in public health, have created new and often very dissimilar realties for women and men. People are all aware that they differ in their anatomy and physiology, but also in more complex traits, such as lifespan (in Italy, 78.8 years for men and 84.1 years for women, respectively) and mortality [27-29]. No conclusive explanation for these new differences is actually demonstrated. An intricate interaction between environmental, social structural, behavioural (i.e. the complex pattern of roles and values that define what is thought as *masculine *and *feminine*) and genetic factors have been suggested as the more probable reason [30-32].

From a genetic prospective, our suggestion based on the studies in Sicilian population supports a female-specific gene-longevity association, by emphasizing the paradoxical role of socio-cultural habits in female longevity [33]. This concerns the HFE gene, the most telomeric HLA class I gene, codifying for a class I α chain, the HFE protein, which seemingly no longer participates in immunity. It has lost its ability to bind peptides due to a definitive closure of the antigen binding cleft that prevents peptide binding and presentation. The HFE protein, expressed on crypt enterocytes of the duodenum, regulates the iron uptake by intestinal cells, having acquired the ability to form complex with the receptor for iron-binding transferring. Mutations in HFE gene are associated with hereditary hemochromatosis, a disorder caused by excessive iron uptake [34,35]. Three common mutations, C282Y, H63D and S65C, have been identified in HFE gene. In particular, the C282Y mutation (a cysteine-to-tyrosine mutation at amino acid 282) destroys its ability to make up a heterodimer with β2-microglobulin. The defective HFE protein fails to associate to the transferring receptor, and the complex cannot be transported to the surface of the duodenal crypt cells. As a consequence, in homozygous people, two to three times the normal amount of iron is absorbed from food by the intestine, resulting in end-organ damage and reducing lifespan. Two other mutations, H63D (a histidine to aspartate at amino acid 63) and S65C (a serine to cysteine at amino acid 65), are associated with milder forms of this disease [34,35].

An association between C282Y mutation and longevity characterizes the Sicilian population studied [33]. In particular, women carriers of C282Y mutation had a higher frequency among the oldest old compared to control women (Table 3). Thus, the C282Y mutation may confer a selective advantage in terms of longevity in Sicilian women. Considering the historical and social context in which the generation of women under study lived, our data seem to propose that the possession of iron-sparing alleles significantly increases the possibility for women to reach longevity. For instance, in Sicily, many pregnancies and an iron-poor diet, consisting mainly in grains, vegetables, and fruits, were still the rule for women born at the beginning of last century. In fact, meat was available for men but not for women; this clearly explains how genetic background also interacts with culture habits [30,31,33].

**Table 3:** Data from our investigations in Sicilian population


**Gene**	**Alleles of genetic variants**	**Centenarians**	**Young controls (< 55 years)**		**P**

		N = 35 females	N = 106 females		

HFE	C282	47 (84%)	132 (0%)		8.3 × 10^-5 ^[33]
	282Y	9 (16%)	0 (0%)		

**Genes**	**Alleles of SNPs or genetic variants**	**Centenarians**	**Young controls (< 55 years)**	**MI patients (< 55 years)**	**P**

		N = 55 males	N = 127 males	N = 105 males	

TLR4	+896A	94 (85.4%)	239 (94.1%)	205 (97.6%)	< 0.001 [49]
	+896 G	16 (14.6%)	15 (5.9%)	5 (2.4%)	

		N = 123 males	N = 136 males	N = 133 males	

CCR5	WT	221(89.8%)	252 (92.6%)	263 (98.8%)	0.00006 [48]
	Δ32	25 (10.2%)	20 (7.4%)	3 (1.2%)	

		N = 96 males	N = 170 males	N = 140 males	

Cox-2	-765 G	122 (63.5%)	240 (70.6%)	232(82.8%)	0.000007 [47]
	-765 C	70(36.5%)	100(29.4%)	48(17.2%)	
5-Lo	-1708 G	180 (93.7%)	302(88.8%)	224(80%)	0.00003 [47]
	-1708A	12(6.3%)	38(11.2%)	56(20%)	0.001
	21 C	176(91.7%)	299(88%)	225(80.4%)	
	21 T	16(8.3%)	41(12%)	55(19.6%)	

**Genes**	**Alleles of SNPs or genetic variants**	**Centenarians**	**Young controls (< 55 years)**	**PC patients (< 55 years)**	**P**

		N = 55 males	N = 125 males	N = 50 males	

TLR4	+896A	94 (85%)	235 (94%)	99 (99%)	0.001 [54]
	+896 G	16 (15%)	15 (6%)	1 (1%)	

Cox-2	-765 G	67 (61%)	176 (70%)	77 (77%)	0.05
5-Lo	-765 C	43 (39%)	74 (30%)	23(23%)	0.0007
	-1708 G	104 (95%)	223 (89%)	77 (77%)	
	-1708A	6 (5%)	27 (11%)	23 (23%)	

		N = 53 males		N = 50 males	

CCR5	WT	95 (89.6%)		97 (97%)	0.03 [53]
	Δ32	11(10.4%)		3(3%)	

**Gene**	**Alleles of SNPs or genetic variants**	**Centenarians**	**Young controls (< 55 years)**	**BF patients (30-60 years)**	**P**

		N = 42 females	N = 42 females	N = 42 females	

TLR4	+896A	81 (96.4%)	78 (92.9%)	76 (90.4%)	0.003 [55]
	+ 896 G	3 (3.6%)	6 (7.1%)	8 (9.6%)	

Our data, showing the relevance of C282Y for women survival to late age, allow adding another piece of evidence to the complex puzzle of genetic and environmental factors involved in control of lifespan in humans. The complex interaction of environmental, historical and genetic factors, differently characterizing the various parts of a country, i.e. Italy, likely plays an important role in determining the gender-specific probability of attaining longevity [30,31,33,36].

### Role of innate immunity genes in longevity: the paradigmatic case of TLR4, CCR5, COX-2 and 5-LO genes

According to evolutionary ageing theories, most of the parameters influencing immunosenescence appear to be under genetic control [32,37,38]. An example is given by the innate immune system, involved in neutralizing infectious agents [39]. It plays a beneficial role until the time of reproduction and parental care. In old age, a period largely not foreseen by evolution, it can determine an opposite and detrimental effect through chronic inflammatory responses ("antagonistic pleiotropy") [38,40]. Genetic pro-/anti-inflammatory variations in innate immune response are, indeed, thought to influence the susceptibility of age-related human diseases, by altering host response to environmental and endogenous stress [41]. Thus, they are able to determine a negative or positive control of inflammation, by affecting both interactions between host and microbes and survival of the individual and attainment of longevity. Furthermore, they appear both to be responsible, at least in large part, for different men and women strategies to achieve longevity, and to contribute to the preferential sex dimorphism of the age-related diseases [30,31,33].

From our investigations in Sicilian population, TLR4, CCR5, Cox2, 5-Lo genes can be considered good examples. They provide an ideal model to understand the different implications of their genetic variants in the risk of age-related diseases, i.e. atherosclerosis and prostate cancer (PC), and reciprocally in increased chance to attain longevity.

TLR4 gene (number accession of GenBank: NM-138554.1) codifies the best understood TLR member involved in recognition of LPS, the prototypic TLR4 ligand, and other exogenous and endogenous (i.e. HSPs, hyaluronic acid, β-defensin-2, ox-LDL, fibronectin and amyloid peptide) ligands. TLR4 activation implies a downstream signaling mediated by several intracellular adaptor molecules and the consequent activation of transcription factors, such as NF-kB. This determines the production of different pro/anti-inflammatory mediators. These lasts, such as IL-10, are produced by the parallel activation of anti-inflammatory pathways to limit the potential tissue damage from excessive activation of the innate immune system [42]. SNPs seem to modulate both TLR4 activity and function. In human, only two SNPs, +896A/G (Asp299Gly; rs4986790) and +1196 C/T (Thr399Ile; rs4986791), have a frequency > 5%. They induce a blunted response to LPS, as first suggested by Arbour et al., and are phenotypically associated to changes in the production of cytokines, principally those carrying the Asp299Gly mutation [43-45]. Accordingly, recent literature data suggest the ability of this SNP to modulate the risk of major age-related diseases [42].

The CCR5 gene (number accession of GenBank: NM-00579) codifies for a G protein-coupled chemokine receptor, which regulates trafficking and effector functions of memory/effector Th1 cells, macrophages, NK cells and immature dendritic cells. CCR5 and its ligands are important molecules in viral pathogenesis. Recent evidence has also demonstrated the role of CCR5 in a variety of human diseases, ranging from infectious and inflammatory age-related diseases to cancer. A notable variant of CCR5 gene is a 32 bp (Δ32) deletion, which causes a frame shift mutation in exon 4 (CCR5Δ32; rs333) and determines stop protein maturation and loss of expression of functional CCR5 receptor [46]. Accordingly, it seems to have a protective role against CVD and other age-related diseases, such as PC. It, indeed, determines a slower progression of atherogenesis or cancerogenesis as a consequence of an attenuated inflammatory response.

COX-2 gene maps in the 1q25 chromosome and codifies for the Cox-2 enzyme involved in the conversion of arachidonic acid to prostaglandins. Polymorphisms regulate its expression and hence prostanoid biosynthesis. In particular, it has been identified a guanine to cytosine substitution at position -765 G/C, located within a putative binding site for the transcription factor Sp1, associated to a reduction in the risk of clinical cardiovascular events. COX-2 is expressed at low levels in most tissues, but its expression enhances under inflammatory stimuli and in inflammatory age-related processes, i.e. atherosclerosis, rheumatoid diseases and cancer [47].

The 5-LO gene maps in the chromosome 10q11.2 and codifies the 5-Lo enzyme involved in the synthesis of LTs. The 5-LO pathway has been associated to atherosclerosis in mouse and human histological studies. Several SNPs have been described. In particular, the - 1708 G/A, -1761 G/A and 21 C/T SNPs in promoter region and exon-1 of 5-LO gene modify the gene transcription or the putative protein [47].

An over-expression of anti-inflammatory CCR5Δ32 variant, +896 G (299Gly) TLR4 allele, -765 C Cox-2 allele, -1708 G and 21 C 5-Lo alleles characterizes male Sicilian centenarians (Table 3) [47-49]. So, male centenarians are people who seem genetically equipped for defeating major age-related diseases. They present SNPs in the immune system genome (i.e. SNPs or other genetic variations, located within the promoter regions of pro-inflammatory cytokines) which, regulating the immune-inflammatory responses, seem to be associated to longevity [30-32]. Furthermore, centenarians are characterized by marked delay or escape from age-associated diseases, responsible for the high mortality in earlier ages. In particular, it has been demonstrated that centenarian offspring have an increased likelihood of surviving to 100 years and show a reduced prevalence of age associated diseases, such as CVD, and lower prevalence of CVD risk factors [1,30-32,50] Thus, genes involved in CVD may play an opposite role in human male longevity. Our data in male Sicilian population confirm this suggestion and emphasize the role of antagonistic pleiotropy in ageing and longevity [51,52]. A high frequency of proinflammatory CCR5wt variant, +896A TLR4 allele, -765 G Cox-2 allele, 1708A and 21 T 5-Lo alleles characterizes male Sicilian patients affected by MI (Table 3) [47-49]. In a recent study, we also found a similar overexpression of these proinflammatory SNPs in male Sicilian patients affected by PC (Table 3). Opposite data were obtained in male centenarians [53,54].

In contrast, female Sicilian centenarians have a different frequency of the alleles of +896A/G TLR4 SNP than that observed in male Sicilian centenarians. In particular, female Sicilian centenarians show an over-expression of the pro-inflammatory +896A TLR4 allele respect to female patients affected by Boutonneuse fever and age-matched controls (Table 3) [55].

On the other hand, pro-inflammatory responses are evolutionary programmed to resist fatal infections. Thus, it is not surprising that the genetic background of people that survive to an advanced age may be protective against infections [55].

Based on our data, we suggest that Sicilian men and women may follow different trajectories to reach longevity. For men it might be more important to control atherogenesis and cancerogenesis, whereas for women it might be more important to control infectious diseases [30,31].

In order to confirm our suggestions on the biological effects of +896A/G TLR4 SNP and its role in the pathophysiology of age-related diseases studied (i.e. MI and PC) and longevity, we recently assessed the levels of IL-6, TNF-α, IL-10 and eicosanoids in LPS-stimulated whole blood samples from 50 young healthy Sicilians, screened for the presence of this SNP. Both pro-inflammatory cytokines and eicosanoids were significantly lower in carriers bearing the +896 G TLR4 allele, whereas the anti-inflammatory IL-10 values were higher [56]. This suggests the ability of the +896 G TLR4 allele to mediate a better control of inflammatory responses induced by chronic stimuli, so likely decreasing the effects of atherogenetic damage and prostate carcinogens.

On the basis of data reported herein, some suggestions can be drawn. First, pathogen load, by interacting with the host genotype, determines the type and intensity of inflammatory responses, according to the pro-inflammatory status and tissue injury, implicated in the patho-physiology of major age-related diseases. Second, adequate control of inflammatory response might reduce the risk of these diseases, and, reciprocally, might increase the chance of extended survival in an environment with reduced pathogen load. Accordingly, a higher frequency of the anti-inflammatory +896 G TLR4 allele has been observed in centenarians [49].

### Cytokine profile: a biomarker for successful ageing

Cytokines are considered key players in maintaining lymphocyte homeostasis [57,58]. Their function is not limited to induce response after an immune insult, but they can modulate the nature of response (cytotoxic, humoral, cell mediated, inflammatory or allergic) or, in contrast, they may cause non-responsiveness and active immune suppression [58]. Furthermore, sequence variations in several cytokine genes, such as IFN-γ and IL-10 genes, have been demonstrated to be associated with successful ageing and longevity [58]. On the other hand, individual changes in type and intensity of immune response affecting life span expectancy and health ageing seem to have a genetic component. A well-preserved immune function characterizing the successful ageing has been found in centenarians [38]. Recent evidence suggests that centenarians seem to be genetically equipped gene polymorphism for overcame the major age-related diseases and polymorphisms in immune system genes involved in regulation of immune responses have been found associated to longevity. In particular, associations between both cytokine gene polymorphisms and longevity, and differential gender longevity in males and females, and reciprocally to age-related diseases have been demonstrated [38,58,59].

Our data in Sicilian population confirm these associations and suggest that differences in the genetic regulation of immune inflammatory processes might explain the reason why some people but not others develop age-related diseases and why some develop a greater inflammatory response than others. In particular, this suggestion seems to be suitable for some SNPs in IFN-γ and IL-10 genes (Table 4) [60-63].

**Table 4:** Cytokine data from our studies in Sicilian population


**Gene**	**Genotypes**	**Centenarians**	**Young controls (< 55 years)**		**P**

		N = 31 males	N = 161 males		

IL-10	-1082GG	18 (58%)	55 (34%)		< 0.025 [61]
	-1083GA	9 (29%)	88 (55%)		
	-1082AA	4 (13%)	18 (11%)		

		N = 72 males	N = 115 males		

IL-10	-1082GG	33 (46%)	32(28%)		0.019 [62]
	-1083GA	34(47%)	64(56%)		
	-1082AA	5(7%)	19(16%)		

		**Centenarians**	**Young controls (< 55 years)**	**MI patients (< 55 years)**	**P**

		N = 52 males	N = 110 males	N = 90 males	

IL-10	-1082GG	25 (48.1%)	26(23.6%)	17 (18.9%)	0.003 [63]
	-1083GA	23 (44.2%)	56 (50.9%)	29 (32.2%)	
	-1082AA	6(11.5%)	28 (25.5%)	44 (48.9%)	

**Genes**	**Alleles of SNP**	**Centenarians**	**Young controls (< 55 years)**		P
		N = 142 females	N = 90 females		
IFN-γ	+874 T	102 (35.9%)	85 (47.2%)		0.02 [60]
	+ 874A	182 (64.1%)	95 (52.8%)		

IFN-γ gene codifies for a cytokine involved in defense against viruses and intracellular pathogens, and in induction of immune mediated inflammatory responses. Its production is genetically regulated. A variable length CA repeat sequence in the first intron of IFN-γ gene has been described to be associated with high IFN-γ production. Furthermore, a SNP, T to A (+874 T/A), at 59 end of the CA repeat region has been described and T presence has been related to high-producing microsatellite allele 2. This SNP coincides with a putative NF-κB binding site, which might have functional consequences for transcription of IFN-γ gene. Thus, this SNP might directly influence IFN-γ production levels associated to CA microsatellite marker [60].

IL-10 gene codifies for IL-10 cytokine. IL-10 is produced by macrophages, T and B cells. It is one of the major immune-regulatory cytokines, usually considered to mediate potent down-regulation of inflammatory responses. IL-10 production, independently on interaction with other cytokine gene products, is generally controlled by several polymorphic elements in the 5' flanking region of IL-10 gene. Multiple SNPs have been identified in human IL-10 5' flanking region and some of these (i.e. -592, -819, -1082) combine with microsatellite alleles to form haplotype associated with differential IL-10 production. These three SNPs in the IL-10 proximal gene region (considered potential targets for transcription regulating factors) might be involved in genetic control of IL-10 production, even if contrasting literature data have been reported. In particular, the homozygous -1082GG genotype seems to be associated with higher IL-10 production respect to G/A heterozygous and AA homozygous genotypes. Furthermore, this SNP seems to be functionally relevant. It has been demonstrated that -1082 A carriers (low producers) seem likely develop a major number of chronic inflammatory diseases [61-63].

Our results demonstrated an increase of subjects carrying the -1082 G IL-10 allele in centenarian men [61-63]. This allele is associated to significantly increased IL-10 production. Conversely, we observed that the frequency of -1082A allele, associated to low IL-10 production, was significantly higher in MI patients (Table 4) [63]. Thus, high IL-10 production seems to be protective vs. MI and a possible biomarker for longevity. People with exceptional longevity have genetic factors (i.e. protective factors for CVD) that modulate ageing processes [63]. This supports the opinion that a genetic background protective against CVD is a component of longevity. On the other hand, our immune system has evolved to control pathogens and pro-inflammatory responses are likely programmed by evolution to resist fatal infections. From this prospective, low IL-10 production is correlated with increased resistance to pathogens. In older ages not evolutionally programmed, increased IL-10 levels might better control inflammatory responses induced by chronic vessel damage and reduce the risk for atherogenetic complications. These conditions might permit to achieve exceptional ages in an environmental with a reduced pathogen load [63].

In contrast, female Sicilian centenarians are characterized by an over-expression of +874 INF-γ allele (Table 4) [60]. The INF-γ production is also influenced by hormonal control fundamentally mediated by 17β extradiol. Hormonal regulation of this cytokine has been suggested to modulate, in part, the ability of estrogens to potentiate many types of immune responses and to influence the disproportionate susceptibility of women for immune-inflammatory diseases. Thus, gene variants representing genetic advantage for one gender might not be reciprocally relevant for the other gender in terms of successful or unsuccessful ageing [60].

The data from Sicilian investigation add another piece to complex puzzle of genetic and environmental factors involved in the control of life span expectancy in humans. Studies on cytokine gene SNPs may promise to individuate a complex network of trans-inactive genes able to influence the type and strength of immune responses to environmental stressors, and as final result, conditioning individual life expectancy [60-63]. On the other hand, we recently suggested the possibility to use cytokine profile as biomarker of successful ageing, by evaluating through Lumines technology cytokine serum levels in 44 Sicilian nonagenarians and 79 control subjects (aged between 30 and 50 years old) [64]. IFN-γ and IL-2 levels are unmodified, suggesting a substantial maintenance of relevant T functions. In addition, a significant increase of IL-12 serum levels was observed. This condition might be associated with the increase of NK cell function with ageing. Furthermore, an increase of IL-13 and a reduction of IL-4 were found. Thus, the maintenance of some effector's mechanisms of immune-response characterizes advanced ages. From a general point of view, our data firstly confirm the age-related remodeling of cytokine network. Furthermore, they underline the presence of unchanged levels of some crucial cytokines useful in preserving key immune function in long-living persons [64].

### Future perspectives

The ageing process and longevity are multi-factorial events. Genetic, epigenetic, stochastic and environmental factors seem to have a crucial role in ageing and longevity. Epigenetic is associated to ageing, as shown in the major number of studies. In particular, ageing is associated to a global loss of methylation state [65]. In addition, tissue-dependent age-related hypermetylation of specific DNA regions have been observed. Thus, it can be concluded that epigenetic age-related modification are stochastic and no linked to specific DNA region, while epigenetic changes linked to specific environmental stimuli are limited in specific DNA region [66,67]. These observations have led to address the research on epigenomics and its implication in ageing and longevity.

Epigenomics is the systematic study of the global gene expression changes due to epigenetic processes, but not to DNA base sequence changes. Epigenetic processes consist in heritable modification that result in a selective gene expression or repression and consequently in phenotype changes [68]. These changes include nucleosome positioning, post-translation histone modifications, action of small RNAs, DNA replication timing, heterochromatinization and DNA methylation [69]. This last one consists in the addition of a methyl group (-CH3) in the carbon 5 of cytosines, particularly in the CpG dinucleotide. This condition particularly concerns the CpG islands (CpGIs), located at the regulatory site of gene promoter regions. Methylation rate is associated to transcriptional regulation. In particular, gene silencing is associated to increase of -CH3 groups on DNA, conversely hypometylation of CGIs is associated to an open chromatin state resulting in gene expression [70].

Although the association between ageing and epigenetic is a real evidence, processes involved are not clear. Certainly, the nutrition affects epigenetic modifications. Nutrients can be active on specific sites. For example, vitamin B12, vitamin B6, riboflavin, methionine, choline and betaine, well known as folates, regulate levels of S-adenosylmethionine and S-adenosylhomocysteine, donor of -CH3 group and methyltransferase inhibitor respectively [71]. Curcumin, resveratrol, polyphenols and flavonoids, phytoestrogen, and lycopene are also considered key nutritional factors both for regulation of enzyme involved in acetylation and deacetylation mechanism and for one-carbon metabolism [71,72]. A diet rich in vegetables and fruit, such as Mediterranean diet, may contain these nutrients. Sicilian centenarians are characterized to observe this kind of diet, as we reported [73]. Since genetic and environmental factors contribute to longevity, it may suggest that epigenetic events associated to the modifications diet-induced are very important for successful ageing processes. Furthermore, several literature data reported a possible link between epigenetic and several age-related diseases, such as cancer, metabolic syndrome, diabetes and neurodegenerative disorders. Stable propagation of gene expression from cell to cell during disease pathogenesis is regulated by epigenetic mechanisms. For example, during the diabetes onset epigenetic changes act on insulin and insulin metabolism regulating the gene coding [74]. In particular, a recent study has demonstrated that human insulin gene and mouse insulin 2 gene expression are under control of epigenetic changes in CpGIs. Insulin non expressing cells are, indeed, methylated in the promoter region of insulin coding gene, while insulin expressing cells are completely demethylated in the same site resulting in insulin gene expression [75]. Another study on monozygotic twin has demonstrated that insulin resistance is also under control of DNA methylation [76]. Alterations in insulin pathway are known to be involved in metabolic disease, such as metabolic syndrome, insulin resistance and type 2 diabetes. Recent data also support the existence of a correlation between these alterations and Alzheimer's disease.

In the light of these observations, the purpose of our future studies will be to evaluate the weight of epigenetic changes in ageing and longevity, using centenarians as super-controls.

## Abbreviations

AD: Alzheimer's disease; BF: Boutonnese fever; CCR5: CC chemokine receptor 5; COX-2: Cyclo-oxygenase 2; CRP: C reactive protein; CVD: Cardiovascular disease; HSPs: Heat-shock proteins; INF-γ: Interferon- γ; IL-6: Interleukin-6; IL-10: Interleukin-10; 5-LO: 5-lipoxygenase; LPS: Lipopolysaccharide; LTs: Leukotrienes; MI: Myocardial infarction; ox-LDL: Oxidized-Low Density Lipoproteins; PC: Prostate cancer; PGs: Prostaglandins; SNPs: Single nucleotide polymorphisms; TLR4: Toll-like-receptor-4; TNF-α: Tumor necrosis factor-α.

## Competing interests

The authors declare that they have no competing interests.

## Authors' contributions

CRB, GA, SB, MB, AM and GCR wrote the first draft. Subsequent drafts were written by CRB, who had the overall supervision of the review processing. All authors edited the paper and approved its final version.
